# Effect of the Applied Fertilization Method under Full Straw Return on the Growth of Mechanically Transplanted Rice

**DOI:** 10.3390/plants9030399

**Published:** 2020-03-23

**Authors:** Jichao Tang, Ruoyu Zhang, Hechao Li, Jun Zhang, Shaoqiang Chen, Bilin Lu

**Affiliations:** 1 Hubei Collaborative Innovation Center for Grain Industry, Agricultural college, Yangtze University, Jingzhou 434025, China; 201872415@yangtzeu.edu.cn (J.T.); zry15897856796@163.com (R.Z.); TUTUTUWAIT@163.com (H.L.); zj15090718972@163.com (J.Z.); CM17362845384@163.com (S.C.); 2 Engineering Research Center of Ecology and Agricultural Use of Wetland, Ministry of Education, Jingzhou 434025, China; 3 Hubei Provincial Key Laboratory of Waterlogged Disasters and Agricultural Use of Wetland (Yangtze University), Jingzhou 434025, China

**Keywords:** straw return, nitrogen fertilizer, combined application, growth, mechanically transplanted rice

## Abstract

This study aimed to improve nitrogen utilization and alleviate the inhibition of straw decomposition during early tillering and the growth of paddy after straw return. Specifically, three different nitrogen fertilizer (base fertilizer) application methods were tested under full straw return: applying the compound fertilizer once (J1), applying the compound fertilizer twice (J3) and applying the ammonium carbonate fertilizer plus compound fertilizer (J2). Full straw return without fertilizer (CK1) and no straw return without fertilizer (CK2) were used as the controls. The results showed that treatment with ammonium carbonate fertilizer combined with compound fertilizer (J2) significantly enhanced straw decomposition, light interception and dry matter accumulation at an early stage of tillering, but reduced tiller occurrence at a late tillering stage. Grain yield was affected due to reduced dry matter accumulation, nitrogen use efficiency and number of effective panicles. There were no significant differences in rice growth, nitrogen use efficiency and grain yield between the one-time or two-time compound fertilizer application methods. In contrast, treatment with ammonium carbonate fertilizer combined with compound fertilizer (J2) under full straw return effectively improved straw decomposition and accelerated the return of green and tillering. In addition, the proportion of ammonium carbonate fertilizer affected the nutrient utilization efficiency and yield at later stages.

## 1. Introduction

As natural supplements containing valuable nutrients and organic carbon (C), crop straw is often incorporated into soils in sustainable agriculture [1]. The incorporation of crop residues, such as straw, can have positive effects on agriculture, including improved nutrient availability and water retention, better soil structures and a less risk of erosion [2]. Straw incorporation can also decrease the amount of nitrogen fertilizer needed [3]. As such, straw return can reduce soil degradation, maintain soil fertility and promote crop production in intensive agricultural systems [4,5,6].

However, straw return can bring about a series of agricultural problems. For example, straw can immobilize nutrients from soil and fertilizers, which inhibit the early growth of rice [2,3]. This may be due to the rapid decomposition of straw and the accumulation of large amounts of harmful substances, which can inhibit rice growth [7]. Moreover, due to the high C/N content in straw [8], much of the nutrient supply supports straw decomposition [9,10,11], rather than promoting rice growth and nutrient absorption [12]. Previous studies have reported that wheat and maize residues associate with low N mineralization, because N immobilization from the soil negatively influences the amount of available N in plants [13,14,15,16]. On the other hand, due to advancements in agricultural mechanization, the slow decomposition rate of large amounts of straw (the annual decomposition of straw plowed into the field is about 60%) results in the accumulation of straw [17,18], which is not conducive to planting and growth. Therefore, it is imperative to accelerate the maturation of straw and reduce the inhibition of early rice growth by straw return. Yan et al. [3] showed that straw mulch, which increased the content of N fertilizer, could significantly reduce the growth inhibition at the early stages of rice growth and increase dry matter accumulation. However, the use of high amounts of N leads to its lower application in subsequent periods, resulting in a shortage of N needed for rice growth, especially in low-fertility paddy soil. At present, there are few studies on alleviating the adverse effects of straw decomposition on growth in the early stages of rice growth; reports on how rice base fertilizer affect straw decomposition are even more scarce. On this basis, we investigated the effects of different types of base fertilizers on straw degradation and rice growth, aiming to alleviate the inhibition of straw decomposition on early tillering and growth of paddy, and to improve nitrogen utilization after straw return. In addition, it needs to be explained that after full return of wheat straw, i.e., wheat straw fully incorporated into the paddy soil through mechanical plowing in the rice-wheat rotation system. This research suggests a reasonable combination of straw return and cultivation for rice-wheat double cropping farmlands.

## 2. Materials and Methods

### 2.1. Experimental Materials

The rice variety was Quanliangyou 681, which was provided by Hubei Quanyin High-tech Seed Industry Company Limited. Ammonium carbonate, compound fertilizers and urea were obtained from Hubei Xinshengyuan Biological Engineering Company Limited, Xinyangfeng Agricultural Technology Company Limited and Hubei Qianjiang Jinhua Run Fertilizer Company Limited, respectively.

### 2.2. Field Site and Experimental Design

From 2017 to 2018, the field experiment took place in the Agricultural Science and Technology Industrial Park of Yangtze University, Huazhong Agricultural High-Tech Industrial Development zone, Jingzhou City, Hubei Province, China (30°22′ N, 112°4′ E). In 2017, only the grain yield was measured, and in 2018, the grain yield and other indices were measured. The soil type was light loam (Kakingski soil texture classification system); the basic physical and chemical properties of the soil in the depth range of 0–30 cm were as follows: a pH of 8.05, an organic matter content of 18.75 g kg^−1^, an alkaline nitrogen content of 90.7 mg kg^−1^, an available phosphorus content of 23.9 mg kg^−1^ and a fast-acting potassium content of 158 mg kg^−1^. The experiment used a randomized block design. The detailed experimental design is shown in Table 1 (J1-3 treatments were conducted under was full straw return). The method of straw return was the full return of wheat straw. The sowing date was May 5, the transplanting date was May 27; the harvesting date was September 22. Seedlings were cultivated in seedling trays and transplanted with a rice transplanter (Kubota Agricultural Machinery Co., Ltd., SPW-28C). The planting density of rice plants was 30 cm × 16 cm. The nitrogen fertilizer was comprised of ammonium carbonate fertilizer (ammonium carbonate fertilizer is a fast-acting nitrogen fertilizer, which can provide fast-acting nutrients. Its nitrogen content is 17.1%), compound fertilizer (N:P_2_O_5_:K_2_O = 15%:15%:15%) and urea (46% N); N was applied at 195 kg hm^−2^ rate in this study, N fertilizer was applied at the basal, tillering (7 days after transplantation, 7 DAT); panicle initiation stage in a ratio of 5:3:2. Base fertilizer was applied 5 days before transplantation and during transplanting. The content of P_2_O_5_ and K_2_O in each treatment was adjusted to 97.5 kg hm^−2^ by using calcium superphosphate (16% P_2_O_5_) and potassium chloride (60% K_2_O); both calcium phosphate and potassium chloride were applied during transplanting. Each experimental plot was 56 m^2^ in area with a 30-cm-wide and 20-cm-high ridge covered with plastic film to prevent water and nutrient penetration between contiguous plots. A total of 15 plots were used in this study.

### 2.3. Testing Index

Rice stem and tiller numbers: The number of seedlings was recorded after transplantation; ten representative plants were selected to calculate the average number of stems and tillers in each plot. The number of rice tillers was investigated every 4 days until the number of tillers stabilized.

The SPAD value of rice leaves: The chlorophyll analyzer SPAD-502 Plus (Konica Minolta Japan) was used to determine the tillering stage, booting stage, full heading stage and maturing stage; the first complete leaf from the main stem was measured. The average of the upper 1/3, middle 1/2 and lower 1/3 of the leaf was taken as the SPAD value for the plant. Each treatment was repeated five times.

Canopy photosynthetically active radiation (PAR) transmission parameters: The tillering, booting, full heading and maturing stages of rice were determined at 11:00–13:00 on a sunny day using the AccuPAR plant canopy line analyzer (Decagon USA) The incident PAR (PAR_T_) was measured at the top of the canopy (30 cm above the top of the plant). The PAR (PAR_R_) was measured at the top of the canopy vertically. The incident PAR (PAR_T_) was measured at the base of the plant canopy.

The straw decomposition rate: Using the nylon mesh bag method, 40.00 g of dried wheat straw was cut to a length of about 5 cm, and then packed into a nylon mesh bag (120 mesh, 35 cm × 25 cm) and buried (5–10 cm) into each plot in the field before transplantation. The mesh bag was removed from the plots, washed with water, dried, and weighed at 5 d, 20 d, 35 d, 50 d, 65 d, 90 d and 120 d after transplantation, and each treatment was repeated three times [19].

The dry matter accumulation and total nitrogen content: At tillering, booting, full head and maturing stages, five plants were selected according to the average number of stems and tillers per treatment, killed at a temperature of 105 °C and dried to constant weight at 80 °C. The dry matter weight was measured and repeated three times. The mature samples were crushed and the nitrogen content in each plant was determined by the ECS 4024 CHNSO Classic Analyzer (Costech, Italy). This procedure was repeated three times.

Yield and yield components: After the maturation of rice, an area of about 5 m^2^ was selected from the central part of each plot for yield measurement. Ten rice plants were selected to investigate the average effective panicle number. At the same time, five plants were selected to determine the number of grains per ear, seed setting rate and 1000-grain weight.

### 2.4. Statistical Analysis

The data were analyzed and plotted using SPSS 17.0 and ORIGIN 2019 software packages. The treatment means were compared using the least significant difference (LSD) test at a probability level of 0.05 (*p* ≤ 0.05).

The decomposition rate of straw in each period was calculated as: (dry weight of initial straw − dry weight of remaining straw) / dry weight of initial straw × 100%

The canopy PAR, transmittance rate (Tr), reflectance rate (Re) and interception rate (In) were calculated as follows:

Tr = PAR_T_ / PAR_I_
Re = PAR_R_ / PAR_I_
In = (PAR_I_-PAR_T_-PAR_R_) / PAR_I_

In the formulae, PAR_I_, PAR_T_ and PAR_R_ are the incident PAR (µmol m^−2^ s^−1^) at the top of the canopy, the incident PAR (µmol m^−2^ s^−1^) at the bottom of the canopy and the reflected PAR (µmol m^−2^ s^−1^) at the top of the canopy.

The apparent nitrogen use efficiency (ANUE) was calculated as follows: (total nitrogen content of plants in application plots with nitrogen − total nitrogen content of plants in application plots without nitrogen)/nitrogen application rate × 100%.

The agronomic use efficiency of nitrogen fertilizer (AE, kg kg^−1^) was calculated as follows: (grain yield in application plots with nitrogen − grain yield in application plots without nitrogen)/nitrogen application rate.

The physiological use efficiency of nitrogen fertilizer (PNUE, kg kg^−1^) = (grain yield in application plots with nitrogen − grain yield in application plots without nitrogen)/(total nitrogen content of plants in application plots with nitrogen – total nitrogen content of plants in application plots without nitrogen).

The nitrogen partial factor productivity (PFP, kg kg^−1^) was calculated as follows: grain yield/nitrogen application.

## 3. Results

### 3.1. Effect of the Applied Fertilization Method on Straw Decomposition Rate

**Figure 1:**
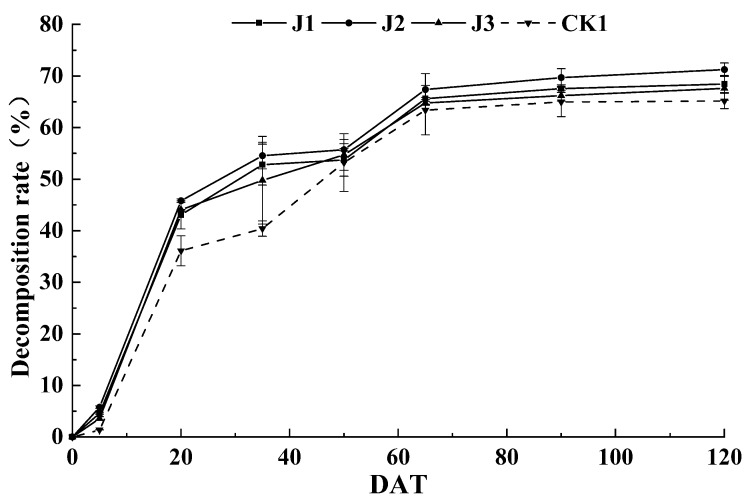
The decomposition rate of straw under the applied fertilization methods. The error bars show the standard error (*n* = 3).

Change of straw decomposition rate with time under different fertilization methods is shown in Figure 1. The straw degradation rate of each treatment showed an increasing trend as the growth progressed, and the decomposition rate was an indicator of earlier and later slowness. The period of fast decomposition occurred before day 20; the decay rate tended to be gradual after day 65 post-transplantation. The straw decomposition rate after non-fertilization treatment was lower than that after fertilization treatment for all time periods. The decay rate after J2 treatment was greater than that after the other treatments for each time period. Within 0 to 20 days after transplantation, the average decomposition rate of J1, J2 and J3 treatments were 0.72 g/d, 0.76 g/d and 0.73 g/d, respectively. Within 65 to120 days after transplantation, the average decay rates of J1, J2 and J3 treatments were 0.02, 0.03 and 0.02, respectively; the straw degradation rate after J2 treatment at 120 d was 3% and 4% higher than those of J1 and J3 treatments, respectively. Taken collectively, these results showed that J2 treatment can effectively increase the straw decomposition rate at early and late stages and increase the degree of straw decomposition. However, there was no significant difference in straw decomposition after the J1 and J3 treatments.

### 3.2. Effect of the Applied Fertilization Method on the SPAD Value in Rice Crop

**Figure 2:**
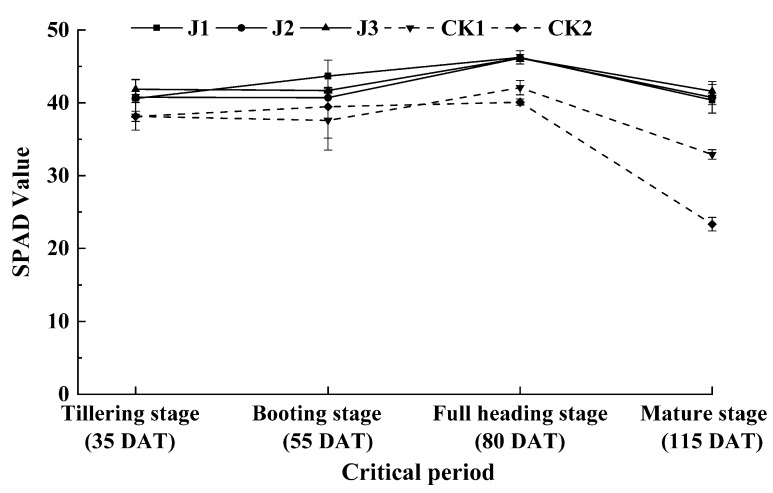
The SPAD value of rice leaves under the applied fertilization methods. The error bars show the standard error (*n* = 3).

Changes in SPAD value of rice leaves with time under different fertilization methods are shown in Figure 2. The SPAD value of each treatment increased first, peaking at the spiking stage, and then decreased as the fertility progressed. The SPAD value in the absence of fertilization treatment was significantly lower than that in the presence of fertilization treatment after straw return. During fertilization, there was no significant difference in the SPAD value after J1, J2, and J3 treatments during the tillering, full heading and mature stages. At the booting stage, the SPAD value after J1 treatment was significantly higher than that after J2 and J3 treatments by 7% and 5%, respectively. In the absence of fertilization treatment, there was no significant difference in the SPAD value between CK1 and CK2 during tillering, booting and full heading stages. During the maturing stage, the SPAD value of CK1 was significantly higher than that of CK2.

### 3.3. Effect of the Applied Fertilization Method on Canopy PAR Transmission Parameters in Rice Crop

**Figure 3:**
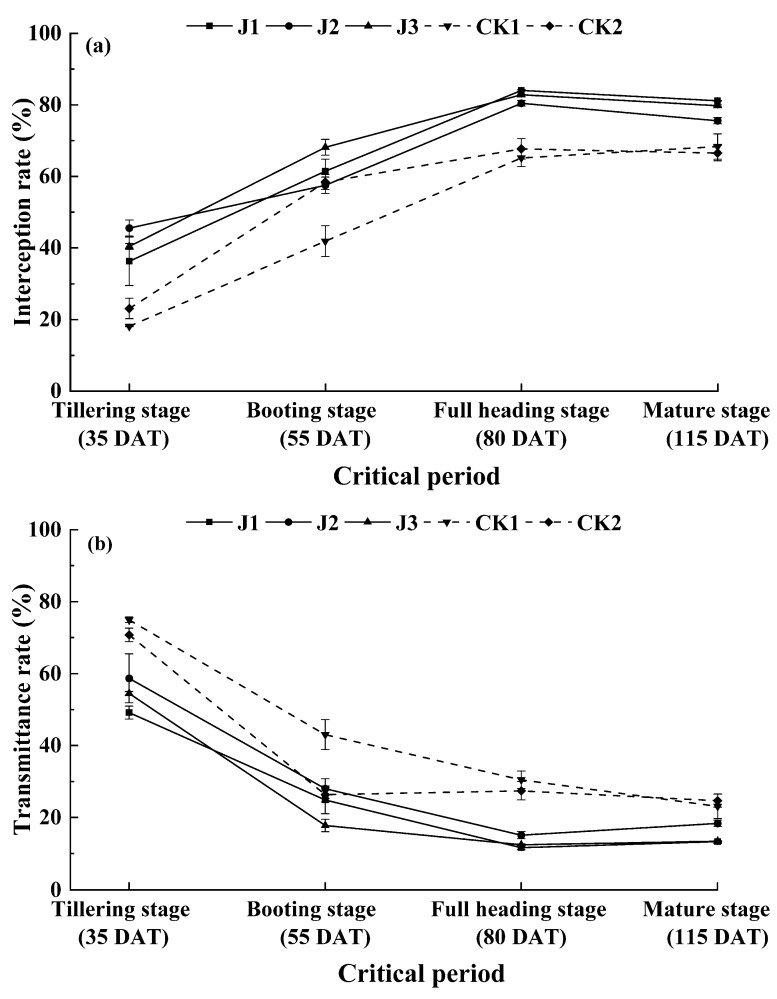

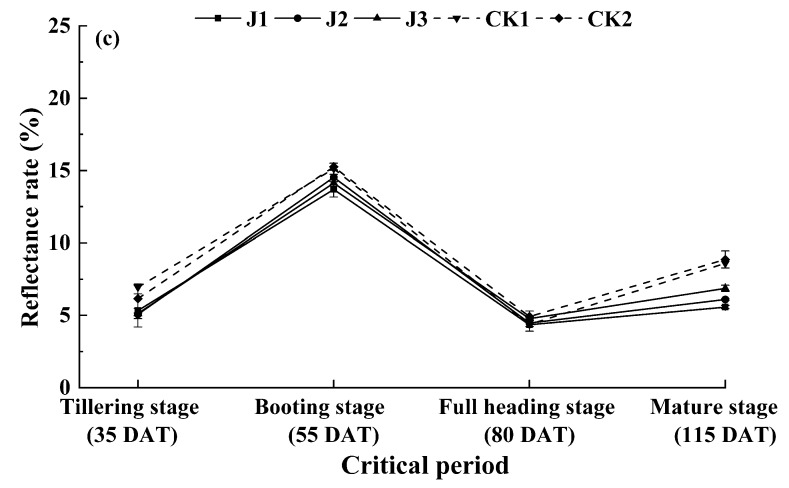
Canopy light interception rate (**a**); transmittance rate (**b**) and reflectance rate (**c**) of rice under the applied fertilization methods. The error bars show the standard error (*n* = 3).

Changes in PAR transmission parameters of rice canopy under different fertilization methods with time are shown in Figure 3. Light interception rate (In) and light transmission rate (Tr) were high, whereas the light reflection rate (Re) was low, indicating that the light interception rate of machine-inserted rice was mainly determined by transmittance, whereas the change in reflectance had little effect on it. With the progression of fertility, the light interception rate of each treatment first increased and then decreased. By contrast, the change in transmittance showed an opposite trend; the change in light reflection rate did not show an obvious pattern. After straw return, light interception rate after fertilization was greater than that after non-fertilization treatment, whereas an opposite trend was observed for the change in light transmission rate; and there was no significant difference in light reflectance between the treatment and control groups. With fertilization, at the tillering stage, the light interception rate after J2 treatment was significantly higher than that after J1 and J3 treatments by 9% and 5%, respectively. By contrast, the light interception rate after J2 treatment was lower than that after J1 and J3 treatments at the other stages. The light interception rate after J3 treatment was higher at the tillering and booting stages than that of J1 treatment, with the change in the booting stage being statistically significant (*p* < 0.05). However, the light interception rates of the panicle and mature stages after J3 treatment were lower than after J1 treatment. In the absence of fertilization treatment, the light interception rate of CK1 was lower than that of CK2 in each period and the difference was significant at the booting stage.

### 3.4. Effect of the Applied Fertilization Method on Stem and Tiller number in Rice Crop

**Figure 4:**
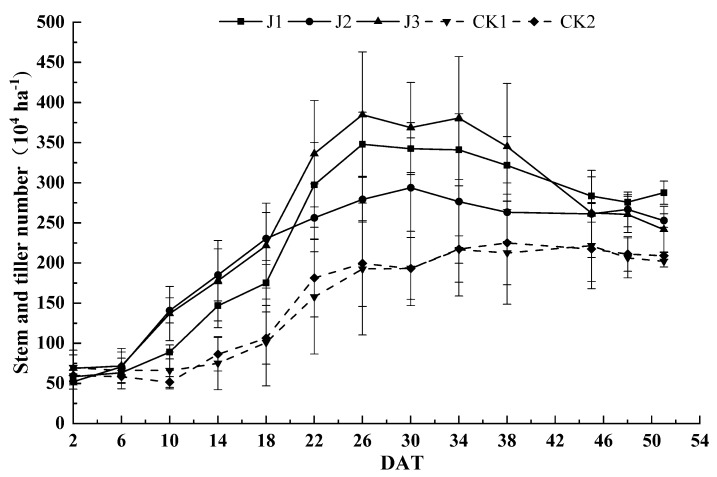
Rice stem and tiller numbers under the applied fertilization methods. The error bars show the standard error (*n* = 3).

Changes of tiller number of rice with time under different fertilization methods are shown in Figure 4. The number of tillers increased slowly with straw return in the absence of fertilization compared with that in the presence of fertilization. The time when seedling numbers maximized was delayed and the peak value was significantly reduced without fertilization compared with that with fertilization. Among the several fertilization treatments, in the early tillering stage, the tillering speed of J2 treatment was the highest and that of J1 treatment was the lowest. However, we observed a slower tiller growth rate in the middle and late stages of tillering after J2 treatment compared with other fertilization treatments. Maximum seedling numbers followed the order of J3 > J1 > J2 and that of J3 was 11% higher than J1 and 23% higher than J2. In the absence of fertilization, the growth rate and maximum seedling number of CK2 at an early stage were slightly higher than those of CK1 after straw return, however, this difference was not statistically significant.

### 3.5. Effect of the Applied Fertilization Method on Dry Matter Accumulation in Rice Crop

Changes in dry matter accumulation of rice with time under different fertilization methods are shown in Table 2. Dry matter accumulation in the tillering, earing and mature stages without fertilization was significantly lower than that after fertilization treatments. At the booting stage, dry matter accumulation without fertilization was significantly lower than that after J3 treatment. Dry matter accumulation was also significantly lower than that after the fertilization treatment from booting to full heading, as well as from full heading to maturation. However, no significant difference in dry matter accumulation was detected between the treatment and control groups from tillering to booting. During fertilization, the dry matter accumulation after J2 treatment was significantly higher than that after J1 and J3 treatments by 16% to 27% at the tillering stage. The dry matter accumulation after J2 treatment at the other stages was lower than that of J1 and J3 treatments, and it reached a significant level at the booting stage after J3 treatment. There was a significant difference between J1 and J3 treatments at the full ear and mature stages; dry matter accumulation after J3 treatment was significantly higher than that after J1 treatment by 10% at maturity, but there was no significant difference after J1 treatment at other stages. There was no significant difference in dry matter accumulation among J1, J2 and J3 treatments from booting to full heading; likewise, there was no significant difference in dry matter accumulation between J1 and J3 treatments from tillering to booting. However, dry matter accumulation from tillering to booting after J1 and J3 treatments was 158% and 217% higher than that after J2 treatment, respectively. The dry matter accumulation of J3 treatment from the spiking to the adult stage was significantly higher by 17% and 24% than that after J1 and J2 treatments, respectively. In the absence of fertilization treatment, there was no significant difference in dry matter accumulation between CK1 and CK2 treatments.

### 3.6. Effect of the Applied Fertilization Method on Nitrogen Use Efficiency in Rice Crop

Nitrogen use efficiency of rice under different fertilization methods is shown in Table 3. The total amount of nitrogen accumulated in non-fertilized plants was significantly lower than that in fertilized plants; there was no significant difference between the two non-fertilization treatments. After fertilization, there was no significant difference in the total amounts of nitrogen accumulation, apparent nitrogen utilization, agronomic nitrogen utilization, physiological nitrogen utilization and partial productivity between J1 and J3 treatments. The total amount of nitrogen accumulated and apparent nitrogen utilization rate after J2 were not significantly different from those after J1 treatment but was significantly lower by 8% and 8% after J3 treatment, respectively. The nitrogen physiological utilization rate after J2 was not different compared with J3 treatment, but it was significantly lower by 34% compared with J1 treatment. The nitrogen agronomy utilization rate and partial productivity after J2 treatment were significantly lower than those of J1 and J3 treatments. For example, the agronomy utilization rate and partial productivity of J2 were significantly lower by 34–40% and 10–13%, respectively, compared with those of J1 and J3.

### 3.7. Effect of the Applied Fertilization Method on Yield and Yield Components in Rice Crop

Rice yield and its composition under different fertilization methods are shown in Table 4. The two-year experiment showed similar regularity in rice yield, taking the results of the 2018 experiment as an example. The yield was significantly higher after fertilization treatment than that after no treatment after the return of straw. During fertilization, the yields of J1 and J3 treatments were 15% and 12% higher than that of J2 treatment; there was no significant difference between J1 and J3 treatments. In the absence of fertilization treatment, the yield of CK1 was 12% lower than that of CK2.

As shown in Table 4, the number of effective ears and grains per year after straw return were significantly higher after fertilization treatment than those after no treatment. After fertilization, there were no significant differences in the number of grains per panicle and 1000-grain weight. The effective panicle number after J1 treatment was 13% and 14% higher than that after J2 and J3 treatment, respectively; there was no significant difference between J2 and J3 treatments. The seed setting rates of J1 and J3 treatments were 4% and 5% higher than that of J2 treatment, respectively, and there was no significant difference between J1 and J3 treatments. In the absence of fertilization treatment, the number of grains per ear of CK2 was 9% higher than that of CK1; there was no significant difference in the yield between CK1 and CK2.

## 4. Discussion

Straw decomposition is beneficial for nutrient absorption by crops and facilitates crop growth in the long term [20]. Previous studies have shown that the application of nitrogen fertilizer in high C/N straw nutrient release and promote crop growth [21]. In this study, the straw decomposition rate after non-fertilization treatment was lower than that after fertilization treatments for all periods tested. Compared with other treatments, J2 treatment more effectively increased straw decomposition rate at early and late stages, thereby accelerating straw maturity. The decomposing rate of non-fertilization treatment was lower than that of fertilization treatments in each stage, whereas the J1 and J3 treatments did not exhibit statistically significant differences in the straw decomposition rate. These results suggest that ammonium carbonate fertilizer combined with compound fertilizer was an effective fertilization method for accelerating straw maturation, presumably because J2 treatment provided sufficient available nitrogen during early straw decomposition.

During the early stages of rice growth, nitrogen deficiency occurs in plants due to the “contending for nitrogen” phenomenon between microorganisms and plants, which can cause growth inhibition [22,23]. In this study, J2 treatment increased the tillering rate at an early tillering stage, thereby significantly increasing dryness later on at the tillering stage. The amount of material accumulated and tillering rate at a late tillering stage was greatly reduced compared with the other two fertilization treatments, and the amount of dry matter accumulated at the booting, full ear and mature stages was reduced. The effective panicle number and seed setting rate were insufficient. As a result, the grain yield was significantly reduced by 12% to 15% compared with the other two fertilization treatments. Compared with J3 treatment, the tillering rate and the maximum seedling number decreased; dry matter content decreased by 9% from the spiking to the adult stage and by 19% at maturation after J1 treatment. In addition, the number of effective spikes increased by 14%, with no significant change in grain yield. The dry matter accumulation of CK1 in each period was lower than that of CK2. As a result, the number of grains per ear was significantly reduced by 8% compared with CK2, leading to a 12% decrease in grain yield.

The efficiency of nitrogen use is an important indicator of nitrogen absorption by rice plants, and it serves as a reference for the application of nitrogen fertilizer in rice fields [24]. In this study, there was no significant difference in nitrogen accumulation between CK1 and CK2. Furthermore, there was no significant difference in nitrogen accumulation and nitrogen utilization between J1 and J3 treatments. Compared with J3 treatment, total nitrogen accumulation and apparent nitrogen utilization rate were significantly reduced by 8% and 8% after J2 treatment, respectively, and the physiological nitrogen utilization rate was 34% lower after J2 treatment than that after J1 treatment. After J2 treatment, agricultural nitrogen utilization rate was 34% to 40% lower and partial productivity was 10% to 13% lower than those of the J1 and J2 treatments. Compared with the other fertilization treatments, J2 treatment greatly reduced nitrogen use efficiency, which was not conducive to the rational use of nitrogen fertilizer.

The SPAD value and photosynthetically active radiation (PAR) are key determinants for plant growth and development. The SPAD value represents chlorophyll content in rice leaves and indirectly reflects the photosynthetic capacity of plants [25,26,27]. Numerous studies have reported that the accumulated biomass of plants is closely related to the amount of photosynthetically active radiation intercepted by the canopy [28,29,30,31]. The photosynthetically active radiation in rice fields is comprised of three components: transmission (light leakage, transmission), interception and reflection. The interception rate of light is one of the determinants affecting light utilization. This study showed that light reflectivity had a negligible effect on light interception. Furthermore, light transmittance was the main factor affecting light interception; light transmittance rate and light interception rate exerted opposite effects. The light interception rate of CK1 was lower than that of CK2 in each period; it was significantly different at the booting stage. Compared with other fertilization treatments, the light interception rate increased from 5% to 9% at the tillering stage after J2 treatment and decreased at the other stages. Compared with J1 treatment, the light interception rate increased by 7% at the booting stage after J3 treatment, but there was no significant difference at the other stages. The SPAD value of J2 treatment was reduced at the booting stage compared with J1 treatment, whereas no significant differences in SPAD value were detected in other developmental stages. These results were in good agreement with the finding of rice growth and nitrogen utilization in this experiment.

Ammonium carbonate, as a fast-acting nitrogen fertilizer, can provide sufficient nitrogen nutrients for microbial maturation at the seedling stage, promote the rapid maturation of straw, accelerate the release of straw nutrients, provide quick-acting nutrients for plant growth and reduce the inhibition of the early growth of rice plants. However, the effects of ammonium carbonate fertilizer are very fast and easily lost, resulting in insufficient nutrients at later stages. In this study, the tillering speed, dry matter accumulation and light interception rate in the early stage of J2 treatment had certain advantages over other treatments. And the straw decomposing rate of J2 treatment was higher than other treatments, which also showed that the early stage of J2 treatment had enough nutrients to support crop growth and improve the crop’s light interception rate. However, these indices and SPAD value and nitrogen utilization rate of J2 treatment showed a certain disadvantage compared with other treatments. These results provided good evidence support for the above speculation. Future studies should focus on optimizing the timing of application and ratio of ammonium carbonate/compound fertilizer to further promote straw decomposition and rapid seedling emergence, as well as to ensure yield gain.

## 5. Conclusions

After full wheat straw was returned to the field, the combination of 25% ammonium carbonate and 25% compound fertilizer as the base fertilizer was conducive to straw decomposition and the rapid seedling emergence of rice.

## Figures and Tables

**Table 1:** Information about the N management used in the experiment.

Treatment	5 Days before Transplantation (N Application Ratio)	During Transplanting (N Application Ratio)	Tillering Stage (N Application Ratio)	Panicle Initiation Stage (N Application Ratio)	Total Nitrogen Application (kg hm^−2^)
J1	0	50%, Compound fertilizer	30%, Urea	20%, Urea	195
J2	25%, Ammonium carbonate	25%, Compound fertilizer	30%, Urea	20%, Urea	195
J3	25%, Compound fertilizer	25%, Compound fertilizer	30%, Urea	20%, Urea	195
CK1	Full straw return without fertilizer				
CK2	No straw return without fertilizer				

**Table 2:** The dry matter accumulation of rice under the applied fertilization methods.

Treatment	Dry Matter Accumulation in Each Growth Stage (t ha^−1^)				Dry Matter Accumulation of Each Stage (t ha^−1^)		
	Tillering Stage	Booting Stage	Full Heading Stage	Mature Stage	Tillering-Booting	Booting-Full Heading	Full Heading-Mature
J1	1.75 ± 0.38 b	4.42 ± 0.80 ab	13.79 ± 0.31 a	20.63 ± 1.17 b	2.67 ± 0.46 ab	9.37 ± 0.58 a	6.84 ± 0.88 b
J2	2.23 ± 0.26 a	3.26 ± 0.26 b	12.37 ± 0.68 b	19.61 ± 0.34 c	1.03 ± 0.12 c	9.10 ± 0.42 a	7.25 ± 0.34 b
J3	1.91 ± 0.12 b	5.19 ± 1.39 a	14.13 ± 0.30 a	22.61 ± 0.42 a	3.28 ± 1.27 a	8.94 ± 1.11 a	8.49 ± 0.12 a
CK1	0.62 ± 0.23 c	3.22 ± 0.77 b	9.26 ± 0.26 c	13.59 ± 0.53 d	2.60 ± 0.68 ab	6.05 ± 0.75 b	4.32 ± 0.34 c
CK2	0.85 ± 0.07 c	2.98 ± 0.81 b	9.57 ± 0.08 c	14.34 ± 0.43 d	2.13 ± 0.75 bc	6.59 ± 0.74 b	4.77 ± 0.35 c

Values represent the mean ± standard error (*n* = 3). Values in columns followed by different letters are significantly different (LSD, *p* < 0.05).

**Table 3:** Nitrogen utilization of rice under the applied fertilization methods.

Treatment	Total N (kg ha^−1^)	ANUE (%)	AE (kg kg^−1^)	PNUE (kg kg^−1^)	PFP (kg kg^−1^)
J1	195.81 ± 5.55 ab	44.49 ± 4.09 ab	19.24 ± 3.15 a	43.93 ± 11.18 a	58.12 ± 2.10 a
J2	187.80 ± 2.14 b	40.38 ± 3.32 b	11.52 ± 1.81 b	28.92 ± 7.12 b	50.40 ± 0.90 b
J3	203.41 ± 8.54 a	48.39 ± 2.67 a	17.36 ± 0.99 a	35.94 ± 2.31 ab	56.24 ± 0.40 a
CK1	109.05 ± 5.61 c	/	/	/	/
CK2	117.28 ± 6.27 c	/	/	/	/

Values represent the mean ± standard error (*n* = 3). Values in columns followed by different letters are significantly different (LSD, *p* < 0.05).

**Table 4:** Rice yield and yield components under the applied fertilization methods.

Treatment	Panicle (10^4^ ha^−1^)	Spikelet Per Panicle	Filled-Grain Percentage (%)	1000-Grain Weight (g)	Actual Yield (kg ha^−1^)	
					2018 Year	2017 Year
J1	263.66 ± 15.41 a	197.60 ± 1.54 a	82.65 ± 0.46 a	29.21 ± 0.12 a	11,332.91 ± 408.79 a	11,048.46 ± 375.94 a
J2	234.26 ± 14.27 b	198.67 ± 0.38 a	78.98 ± 0.18 b	28.82 ± 0.09 ab	9827.76 ± 174.93 b	9598.16 ± 243.71 b
J3	230.56 ± 13.30 b	198.09 ± 13.50 a	84.11 ± 1.30 a	28.79 ± 0.16 ab	10,966.92 ± 77.81 a	11,016.03 ± 139.01 a
CK1	190.74 ± 15.47 c	179.73 ± 5.37 b	78.75 ± 2.71 b	28.64 ± 0.24 bc	7580.80 ± 221.63 d	7927.13 ± 132.24 d
CK2	208.10 ± 3.13 c	195.83 ± 7.33 a	76.64 ± 2.03 b	28.34 ± 0.35 c	8658.96 ± 163.43 c	8748.14 ± 201.36 c

Values represent the mean ± standard error (*n* = 3). Values in columns followed by different letters are significantly different (LSD, *p* < 0.05).
